# The Physicians’ Visiting List

**Published:** 1881-12

**Authors:** 


### THE PHYSICIAN'S VISITING LIST.

This little work for 1882 has just been issued from the press of Linsay & Blakiston, Philadelphia. Something of the kind is indispensable to the physician who wishes to keep his cases well in hand, and to retain a record for future reference. The pages in this book are so prepared and arranged that any conceivable case may be recorded.

It also serves as an appointment book. It is gotten up in good style and convenient form, and is the best thing of the kind that I have seen.

